# Diabetes Patients’ Acceptance of Injectable Treatment, a Scientometric Analysis

**DOI:** 10.3390/life12122055

**Published:** 2022-12-07

**Authors:** Ileana Pantea, Nadinne Roman, Angela Repanovici, Daniela Drugus

**Affiliations:** 1 Faculty of Medicine, Transilvania University of Brasov, 500036 Brasov, Romania; 2 Faculty of Product Design and Environment, Transilvania University of Brasov, 500036 Brasov, Romania; 3 Faculty of Medicine, University of Medicine and Farmacy Grigore T. Popa, 700115 Iasi, Romania

**Keywords:** diabetes, injectable treatment, adherence, compliance, diabetes mellitus, insulin

## Abstract

Diabetes is a condition associated with multiple systemic secondary risk factors, besides pancreatic dysfunctions, affecting the population worldwide and with high costs impacting the healthcare systems. This paper aims to identify the major issues in patients’ adherence to injectable diabetes treatment. After the interrogation of the Web of Science database, a scientometric map was generated, from which six directions of approach were identified as essential factors influencing the patient’s adherence. These directions yielded clusters of related articles. Glycemic control with the endocrinology metabolic implications, lifestyle adjustments, the healthcare services, medication therapy algorithm, healthcare services digitalization and healthcare policies seem to have a major impact on injectable diabetes therapy and patient adherence. Further research on every one of the six directions is needed to identify the potential of increasing injectable treatment adherence in diabetes patients.

## 1. Introduction

Type 1 diabetes is characterized by a lack of or near-lack of b-cells, so insulin is necessary for people with this type of diabetes. In addition to hyperglycemia, hypoinsulinism may contribute to other metabolic disorders such as hypertriglyceridemia and ketoacidosis, as well as potentially life-threatening tissue catabolism [1]. Most individuals with type 1 diabetes should be treated with multiple daily injections of insulin or a continuous infusion of subcutaneous insulin. Type 1 diabetes patients should be educated on how to match their mealtime insulin doses to their carbohydrate intake, fat and protein content, and anticipated physical activity [2,3]. Unlike patients with type 1 diabetes, adults with type 2 diabetes are characterized by a disease with progressive dysfunction of beta cells that evolve at a given moment with the need for insulin administration. For patients with type 1 diabetes, the treatment of insulin in multiple doses is mandatory from the diagnosis; these patients have no treatment alternatives. Therefore, they must accept this injectable treatment. For adults with type 2 diabetes, first-line treatment depends on comorbidities, patient-centered treatment factors, and management needs, but will generally include metformin and a comprehensive lifestyle modification [4,5]. Other medications can be used as diabetes therapy, such as sulfonylureas or insulin glargine, in those with an increased risk of cardiovascular or renal side effects [6,7]. A combination of early treatment options can help extend the time to treatment failure. If there is evidence of ongoing catabolism (weight loss), if symptoms of hyperglycemia are present, or if A1C levels (>10% (86 mmol/mol)) or blood glucose levels (300 mg/dL (16.7 mmol/L)) are very high, the treatment delay must be overwatched. The cost of diabetes medications has grown dramatically over the past two decades, followed by an increase in caregivers and family burden [8,9]. Medication costs can be a major source of stress for people with diabetes and lead to poor medication adherence [10]. In some cases, strategies to reduce costs may improve adherence [11], and initiation of insulin therapy earlier may improve the health and well-being of patients with diabetes. However, even well-controlled diabetes can become more difficult to manage over time [12,13]. Unfortunately, both patients and clinicians are often reluctant to initiate or increase insulin treatment, a phenomenon sometimes referred to as psychological insulin resistance (PIR) [14,15,16,17]. Clinicians may delay starting treatment until it is “necessary” only after alternative therapies have been attempted, and glycemic control has failed to be achieved or maintained by physical control [18]. For patients with type 2 diabetes, precisely due to the fact that injectable treatment is necessary for specific stages of the disease’s evolution, along with the appearance of severe complications, it is essential to know all the factors that delay the initiation of this type of treatment. Initiating the injection treatment as early as possible brings a significant benefit in managing the evolution of the patient with type 2 diabetes with multiple long-term comorbidities. Indeed, the evolution of patients with type 2 diabetes since diagnosis involves a series of non-pharmacological measures (diet and physical activity) and pharmacological with oral medications. However, precisely the moment of decision on the initiation of injectable treatment when all other solutions no longer give results is the subject of our analysis with the help of the scientometric method. Hence, our research aims to find the critical points in initiating and decision-making regarding injectable treatment in the doctor–patient team with type 2 diabetes [17,18].

Electronic registries or communicating databases regarding the patient’s disease history and paraclinical test results seem to be a valuable tool in further diabetes treatment management [19]. For diabetes as a chronic and progressive disease, both the costs of the drugs, but also the costs of the total medical services received by the patient in the medium and long term, should be taken into account in making the treatment decision. Thus, a complete medical record, alongside patient education, are essential to be received, and respectively given, by each specialist for accurate treatment option decision-making [19,20].

Digitization and the use of remote monitoring and control methods are newer and invaluable resources in the treatment of diabetes patients. These tools allow the monitoring of behavior in addition to physiological data by wearable sensors and telephone applications [21]. In the management of diabetes, the processes and methods of involving the patient in making medical decisions were discussed and emphasized, but the results showed that only patients who are open to making decisions by mutual agreement have good therapy adherence, because, otherwise, the results of the therapy did not improve [22,23]. Thus, the medical specialist, as well as the health systems, find themselves facing many diabetes therapy and care options, but also with a heterogeneity of the therapy scheme, monitoring methods, and patient adherence to treatment.

Given the complexity of the disease and the presence of multiple disabling chronic complications, injectable treatment is mandatory for patients with type 1 diabetes, and for patients with type 2 diabetes, injectable therapy has specific indications.

Therefore, this review aims to identify the adherence of diabetic patients to long-term injectable treatment and the socioeconomic implications of these therapies.

## 2. Materials and Methods

A scientometric overview of a field of research is a valuable source of input for conducting systematic reviews, particularly when relevant and up-to-date systematic reviews are not readily available or accessible [19].

Scientometry is the quantitative analysis of the generation, propagation, and use of scientific information, and it allows for the identification of large and emerging trends in scientific research in a specific field of study, based on statistical analysis of databases and the application of qualitative filters (themes, keywords, journals).

Simultaneously, it enables the examination of the evolution of research over time as well as the geographical and organizational distribution of scientific production. The authors, their works, the bibliographic references, and the citations received are the primary scientific data used for scientometric research.

Scientometric analyses enable the identification of the most current topics and research topics in each field, as well as the most cited papers and authors, addressing a specific issue. Can one determine which countries, institutions, and journals have a greater influence on the development of science in a specific field and how interest in a specific scientific discovery varies over time?

The review of the literature should include all the major themes and subtopics identified within the study’s general topic. The traditional review of the scientific literature is frequently haphazard and can result in biased conclusions; it frequently analyzes studies with unrepresentative samples in an unsystematic and uncritical manner. As a methodology for scoping, it is used to respond to broad and specific questions about a specific topic.

A thorough search is conducted, which may be limited by time or scope constraints. It may entail more structured searches than usual.

We performed a research study based on direct interrogation of the scientific literature, based on the Web of Science (WoS) database.

We have established that the main research items are composed of “decision making”, “diabetes Mellitus” and “insulin”. The advanced search option was selected for interrogation, and the query terms were set to “All fields” with the Boolean “and” value.

The query for the study was: “((ALL = (decision making)) AND ALL = (diabetes Mellitus)) AND ALL = (insulin)”. After the initial interrogation, a total of 232 articles were shown.

The study’s article inclusion criteria were based on publication years (2017–2022), access policy (open access), and article type (original research). The exclusion criteria were based on inclusion criteria values that were outside the established range. A total of 46 articles were examined for our study after applying all of the inclusion and exclusion criteria. The methodology of the scientometric research is depicted in Table 1.

Primary data were downloaded as plain text files from the Web of Science—WoS database. The results were examined using VOS Viewer software version 1.6.16 [20], which allows scientific mapping to analyze the content of titles and abstracts of scientific publications. Thus, the VOS viewer term identification function was used to systematically identify key terms in the database (co-word analysis) and organize large amounts of text in a semantic map, ignoring the elements related to the structure of abstracts and copyright statements that might be included.

For assessing the impact and research directions, we have used the scientometric method [19,21]. For the interpretation, of cluster formation, we have used Vos Viewer. The data file from WoS was exported as a plain text file and was uploaded into VOS Viewer [20].

## 3. Results

VOSviewer is the name of the computer program that we used to create and view bibliometric maps. The program is available to the bibliometric research community for free (www.vosviewer.com, accessed on 10 August 2022) [22].

VOSviewer can be used to build maps of authors or journals based on co-citation data or maps of keywords based on co-occurrence data. The program includes a viewer that allows users to examine bibliometric maps in great detail. VOSviewer can display a map in a variety of ways, each highlighting a different aspect of the map [23].

**Figure 1:**
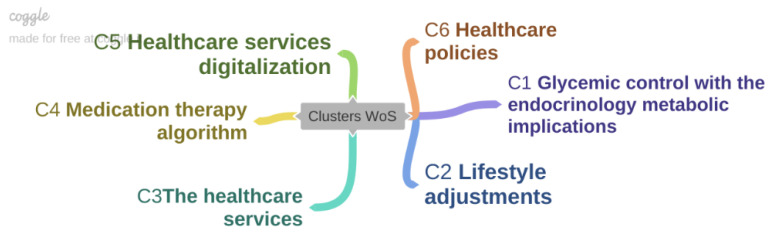
Clusters of research directions.

The interrogated refined database was introduced in VOS Viewer and a scientometric map has been created based on keywords and occurrences of the keywords, to create a relationship between keywords. The results of the scientometric analysis are depicted in Figure 1.

The cluster density view is similar to the regular density view, except that the density is clustered.

Each cluster of items has its own display of items. The cluster density view is especially useful for generating an overview of how items are assigned to clusters and how clusters of items are related to one another [23].

**Figure 2:**
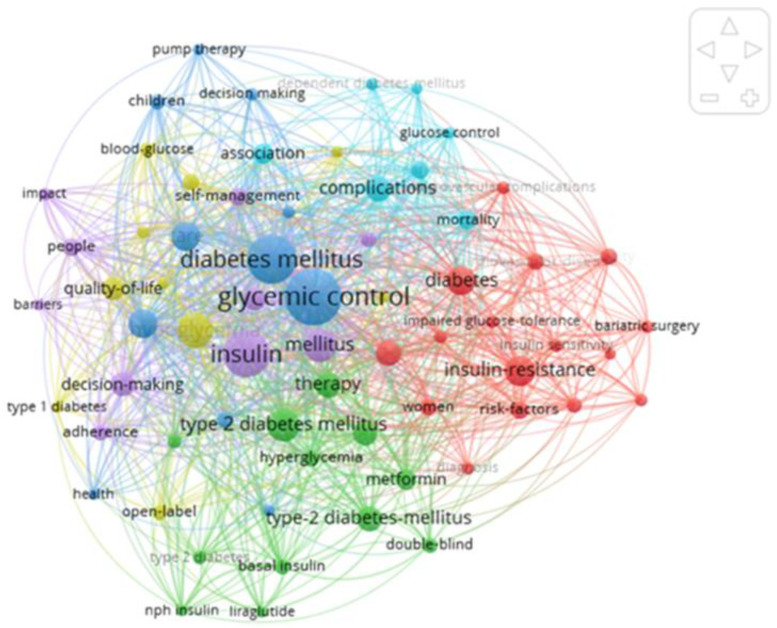
Scientometric map.

A total of 1502 keywords was identified from the total of 232 articles. From those, a threshold of a minimum of 10 occurrences was set. The threshold was met by 203 keywords from which the map was created, depicted in Figure 2. The 203 keywords were divided into 6 clusters, based on occurrence in the screened articles.

The articles result from each cluster are synthesized within the following pieces of review.

### 3.1. Cluster 1—Glycemic Control with the Endocrinology Metabolic Implications

Analyzing this direction, the following findings resulted:

(1) Sapkota et al. realized one analysis of 52 studies carried out between 2000–2013 suggested that diabetic patients’ medication adherence had beneficial effects on their health status. Still, a small number of patients identified the beneficial intervention for glycemic control [24].
(2) Personalized diabetes management therapy combined with patient preferences suppose the administration of a reduced number of doses, minimal requirements for the preparation of injectable products, good glycemic control, and minimal side effects [25].
(3) The follow-up of diabetic patients with religious habits (such as Ramadan) is mandatory to prevent complications. The follow-up could be facilitated by the devices that can record and send data related to blood glucose levels and insulin doses from the patient to the specialist doctor and vice versa [26].
(4) Treatment options must aim at avoiding hypoglycemia and major vascular complications [27].

### 3.2. Cluster 2—Lifestyle Adjustments

Analyzing this direction, the following findings resulted:

(1) Studies show that 40% of diabetic patients consider diet and lifestyle changes important in managing the disease, and 60% consider self-monitoring of blood sugar levels and check-ups with a specialist doctor to be determining factors in the evolution of the disease [26].
(2) The management of the diabetic patient involves: educational self-management, lifestyle modification, and encouraging the patient in making decisions [28].
(3) The personalization of diabetic patient management is a widely debated problem currently within multidisciplinary teams. The main objectives in personalized management are: (a) the patient’s lack of motivation, which leads to a lack of therapeutic compliance and adherence; (b) lack of information regarding patients’ preferences; and (c) the main target is represented by the decrease in glycosylated hemoglobin and the risk of myocardial infarction after 10 years of development of diabetes [29].

### 3.3. Cluster 3—The Healthcare Services

Analyzing this direction, the following findings resulted:

(1) Hypoglycemia is a dreaded complication for any diabetic patient. Avoiding the hypoglycemic episodes can be achieved by training the patient’s caregivers in quick decision-making, to correct the critical situations that affect the state of health. Additionally, the patient’s own previous experiences can be of great help in solving the hypoglycemic occurrence [30].
(2) Diabetics with cognitive impairments, by proving difficulties in adjusting the treatment doses and the occurrence of hypoglycemia episodes, represent essential risk factors in optimizing the therapeutic scheme and keeping the condition under control [31].
(3) Deficiencies in doctor–patient communication are followed by clinical inertia in managing the proposed objectives. The effectiveness of this communication requires: establishing the objectives to be achieved; identifying the most important complications for the patient; identifying the reasons for non-adherence to the treatment; and granting acceptable compromises to the patient in achieving the objectives. Several other factors can improve communication. These involve prolonging visits to the doctor, avoiding the predominant presentation of the adverse effects of the treatment (weight gain, hypoglycemia), and not presenting insulin as a punishment applied to the patient [32].
(4) Doctor–patient communication can help to eliminate many patients’ dilemmas regarding fear of injections and disease self-management [28].

### 3.4. Cluster 4—Medication Therapy Algorithm

Analyzing this direction, the following findings resulted:

(1) The titration algorithm of daily insulin doses seems to be a solid reason for adhering to the treatment. Patients prefer simple algorithms differently from those doctors who prefer algorithms recommended by clinical guidelines. Treatment adherence is strongly influenced by the patient’s preference [9].
(2) Telephone conversations between patients and medical staff are essential for identifying the factors that determine suboptimal treatment adherence of diabetic patients. Through these types of discussions, the lack of disease control due to adequate nutrition and physical effort was identified in 25% of patients who do not want to change these factors [33].
(3) Diabetic patients’ conversations with pharmacists led to the improvement of HbA1c values in elderly diabetic patients [34].
(4) The ideal treatment with insulin must ensure optimal glycemic control, have minimal adverse reactions, be administered orally, and have a low cost [35].
(5) Adherence to insulin treatment seems to be preferential for certain types of insulin: insulin analogs and premixed insulins seem to be preferred by patients [36].
(6) For diabetic patients with mental illnesses, it should be considered that the medication administration shall not cause weight gain and prove a reduced risk of hypoglycemia. Considering these aspects, the first option is suggested to be metformin, whereas the second line of treatment could include GLP1 agonists or DPP4 inhibitors, sulfonylureas, and ultimately insulin. The administration of injectable products with weekly administration seems to be the ideal solution for these patients [37].
(7) Adherence to the treatment is influenced by several factors: weekly administration (GLP1 agonists), presence of injection systems (pens) prefilled with a single dose; important side effects are weight loss and reduced risk of hypoglycemia [38].
(8) Self-interruption of insulin treatment occurs in 20% of patients. Non-adherence to treatment is due to forgetting the daily injections, especially when more doses are needed, and the short duration of the disease [39].
(9) The administration of various new antidiabetic therapies (DPP4, SGLT2 inhibitors) shall be conducted with caution and not on a large scale, due to the short- and long-term effectiveness uncertainty and due to possibly severe side effects [40].
(10) Adherence to antidiabetic medication can be improved if it is cautiously patient tailored and targets all the key factors specific to each individual [41].

### 3.5. Cluster 5—Healthcare Services Digitalization

Analyzing this direction, the following findings resulted:

(1) The use of digital applications for the control of various biological parameters proved to be a useful method in strengthening glycemic control in diabetic patients [42].
(2) Real-time blood glucose monitoring (RT-CGM) ensures a significant reduction of HbA1c compared to self-monitoring of blood glucose (SMGB) in diabetic patients. For severe hypoglycemia, the two monitoring systems did not show significant differences; instead, the costs of the care systems and the health benefits of RT-CGM are much higher [43]. The continuous monitoring of blood sugars demonstrated a decrease in HbA1c between 0.53–1% and hypoglycemia episodes by 38% [44].
(3) For patients with type 1 diabetes, the development of a mobile application such as SOINS DM type would bring significant benefits in the diabetic patient’s life. SOINS DM is a new application with interactive visualization between type 1 diabetes, patients, and the doctor, which facilitates blood sugar monitoring, adjusting insulin doses, physical activity, and diet. This way, a type of personalized feedback could be achieved for this specific type of patient [45].
(4) The presence of a mobile application, using a mobile health technology, with several components such as mobile phones and different sensors, can be beneficial by offering practical advice on monitoring treatment and adjusting insulin doses and optimal blood sugar levels [46].
(5) An algorithm for type 2 diabetes management was developed to provide practical evidence-based guidance for treatment. Additionally, the American Association of Endocrinology has developed a guide with practical recommendations regarding the use of advanced technology in monitoring diabetic patients, allowing nursing staff to make appropriate adjustments to treatment, further enhancing the diabetes technology usability to improve the efficiency and effectiveness of clinical decision-making [47].

### 3.6. Cluster 6—Healthcare Policies

Analyzing this direction, the following findings resulted:

(1) Health insurance systems through the imposed regulations are a determining factor in the inertia of insulin therapy initiation. The pharmaceutical industry is frequently a central source of information regarding new products, therapies, and treatment options. Research suggests that physicians are susceptible to the pharmaceutical industry and interactions with pharmaceutical sales representatives and that these influence prescribing practices. Thus, in the countries of Central and SE Europe, these barriers appear between doctors and their patients and the health insurance systems [48,49].
(2) The implementation of a special patient decision aid (PDA) program, together with physicians, especially concerning poorly controlled diabetic patients, by favoring the decision-making process to enhance treatment and to avoid complications, seemed to be beneficial. This dedicated program includes information about new therapies, their beneficial and adverse effects, doses, and ways of administration [50].
(3) The multidisciplinary team that cares for diabetic patients should address several lines of action: the promotion of supportive relationships, strong commitment, leadership capacity, and diversity in expertise. The establishment of a team consisting of physiotherapists, dieticians, psychologists, and primary care givers seems to be a successful solution in diabetic patients’ management [51].
(4) The high cost of managing episodes of mild hypoglycemia for health systems represents a significant impediment; therefore, avoiding such situations would bring important economic benefits [52].
(5) Until now, the costs regarding the implications of hypoglycemia on work productivity have not been estimated because realistic questionnaires have not been used [44,47,50,53,54,55].

## 4. Discussion

Patient-centered management involves avoiding episodes of hypoglycemia, patients’ treatment preferences, and medication related to their multiple complications. For injectable treatment, this means administration of a reduced number of doses, proper glycemic control, and minimal adverse effects [26].

Recent reviews on this topic suggest that several factors, such as glycemic control, weight changes, treatment dosing frequency and type of administration, gastrointestinal adverse effects, and hypoglycemia, are influencing patients’ behavior, either as motivators or as inhibitors for treatment adherence [56,57]. Mild and moderate hypoglycemia negatively impacts treatment adherence if they occur with a frequency greater than two times per month. Commitment to treatment decreases as the complexity of the treatment increases. Previous research results, similar to our study, also suggest that patient education and good communication between caregivers and diabetics are essential for therapy adherence and sustained disease control.

On the other hand, an individual’s physical barriers seem to be another negative influencing factor regarding diabetes injection therapy adherence. The side effects of the injection were associated with local infection, skin injury, and pain. Alongside these, psychological barriers such as fear and loss of control were reported. Additionally, social barriers were encountered, considering the frequency of daily treatment [58,59]. Patients’ adherence to treatment relies on informing them about the proposed blood glucose lowering targets, as well as possible complications that can arise while the disease develops. In addition, the patient should be encouraged and educated to make informed decisions. The personalization of diabetic patients’ guidance concerns multidisciplinary teams, while personalized management involves identifying the patient’s lack of motivation, preferences, and correct information regarding the risk of fatal cardiovascular complications after ten years of diabetes evolution [31].

The introduction of digital applications is an effective method to help the patient in consolidating the glycemic balance by communicating with the diabetologist through these applications. These monitoring methods are either in real-time (RT-CGM) or through self-monitoring (SMGB), allowing for avoiding episodes of hypoglycemia and better disease control, lowering the burden of healthcare [38]. The identification of advanced technological systems for continuous blood glucose monitoring and the presence of insulin pumps had a beneficial effect, especially on patients with type 1 diabetes. Although the population with type 2 diabetes is much larger, it did not benefit to the same extent from these devices. Hence, it is necessary to identify the tools and bring about these technologies in this population to avoid long-term complications due to defective monitoring of these patients. The costs of diabetic patient management have a significant impact on public health systems, especially in terms of managing hypoglycemia episodes [54]. While the costs concerning hypoglycemia’s impact on work productivity have not been estimated until now, there is a need for prevention solutions [55].

It is critical to comprehend patients’ perceptions of obstacles to insulin introduction as early in the course of the disease as feasible. Healthcare professionals may be able to effectively convince patients to begin insulin therapy earlier by knowing these issues as soon as possible and delivering focused, scientifically accurate information to offset unfavorable or inaccurate impressions. Once on insulin, patients may be able to have a positive experience with the medication and feel more satisfied with their care [51]. Given the significant correlation between treatment satisfaction and medication compliance, improving treatment satisfaction is especially important since we are talking about lifelong treatment [52,56]. To a lesser extent, patients receiving insulin are less satisfied with the treatment than those treated with oral antidiabetics. For people with diabetes, injectable insulin treatment is a sign of their health’s severe deterioration. These patients usually have a long disease evolution and portray many complications. Among the diabetes complications, ulcers of the lower limbs are associated with the lowest degree of satisfaction with the treatment. Therefore, therapy adherence is also negatively influenced [52]. It might be difficult for some people who have misunderstandings about starting insulin therapy to change their minds, especially if those misconceptions have their roots in the experiences of family members who have diabetes (often attributed to insulin use). Therefore, in cases of certain patients, their own concerns and beliefs have much more influence than their doctor’s guidance. When dealing with the doubts expressed by these patients, healthcare professionals may need to be even more proactive. If they don’t have the time to address all their patients’ questions directly, they may need to send them to trained diabetes educators. In most health systems, patients frequently receive education from doctors as much as from other medical staff such as nurses, dietitians, diabetes educators, and employees from official diabetes training programs. It is necessary to conduct further studies on the impact of other healthcare providers, such as nurse educators, who frequently play a significant role in patient care.

The results obtained suggest that scientometric methods enable rapid and efficient analysis of research directions generated by scientific production in the field, making a significant contribution to improving disease approaches and long-term treatments for patients with diabetes.

The scientometric analysis must be interpreted considering the research’s limitations. First, the results are restricted to publications (articles and conference papers) published between 2011 and 2021 and indexed in the Web of Science database. This scientometric analysis, however, has enabled us to identify the key players and research directions in the field in recent years. The study’s findings show that many of the previous interests are still relevant today.

## 5. Conclusions

The scientometric analysis must be interpreted considering the research’s limitations. First, the results are restricted to publications (articles and conference papers) published between 2017 and 2022 and indexed in the Web of Science database.

This scientometric analysis, however, has enabled us to identify the key players and research directions in the field in recent years. The study’s findings show that many of the previous interests in the field are still relevant today. Quantitative measurement, on the other hand, does not allow for the evaluation of the quality of scientific work. However, the scientometric method provides an overview of the research in the field.

Glycemic control with the endocrinology metabolic implications, lifestyle adjustments, the healthcare services, medication therapy algorithm, healthcare services digitalization, and healthcare policies seem to have a major impact on injectable diabetes therapy and patient adherence. Integrated patient management is very important also. The development of technologies that assist the patient’s treatment plan, dosage reporting tools as well as the digitized glycemic result report, supports the specialist in following up with the patient and especially in real-time monitoring of the patient’s evolution. All these facilities are reflected in the costs of medical services, bringing a higher financial return, and achieving much greater benefits for the patient. Qualitative evidence has highlighted individual, healthcare professional, and system-level barriers as reasons for delaying the initiation of insulin therapy to improve glycemic control in people with type 2 diabetes. In recent years, glucagon-like peptide- 1 (GLP 1) analogue injectable treatment for type 2 diabetes has been introduced to overcome some potential barriers associated with insulin medication initiation and adherence, such as weight gain and injection frequency and hypoglycemia.

Further research on every one of the six directions is needed to identify the potential of increasing injectable treatment adherence in diabetes patients.

## Figures and Tables

**Table 1:** Scientometric study stages.

No.	Steps	Description
1	Formulation of the problem	Mapping, bibliometric analysis of publications using descriptors, and research direction identification.
2	Research criteria	Subject: “((ALL = (decision making)) AND ALL = (diabetes Mellitus)) AND ALL = (insulin)”
3	Database used for research	Claryvate analytics, WEB OF SCIENCE—WOS Accessed on
4	Eligibility criteria	Filter 1: years of publication (2017–2022)
		Result: 237 documents.
		Filter 2: articles
		Filter 3: English
		Filter 4: open access
		Result: 46 documents.
5	Data extraction	Bilingual format
6	Analysis and synthesis of results	Qualitative (descriptive) and quantitative (bibliometric) using VOS Viewer
7	Discussions	Analysis of the data gained

## Data Availability

Not applicable.
